# A molecular approach combined with American Thyroid Association classification better stratifies recurrence risk of classic histology papillary thyroid cancer

**DOI:** 10.1002/cam4.1857

**Published:** 2018-12-14

**Authors:** Alexander J. Lin, Pamela Samson, Todd DeWees, Lauren Henke, Thomas Baranski, Julie Schwarz, John Pfeifer, Perry Grigsby, Stephanie Markovina

**Affiliations:** ^1^ Department of Radiation Oncology Washington University School of Medicine St. Louis Missouri; ^2^ Division of Biomedical Statistics and Informatics Mayo Clinic Scottsdale Arizona; ^3^ Division of Endocrinology, Metabolism and Lipid Research, Department of Internal Medicine Washington University St. Louis Missouri; ^4^ Alvin J Siteman Cancer Center Washington University School of Medicine St. Louis Missouri; ^5^ Department of Pathology and Immunology Washington University School of Medicine St. Louis Missouri; ^6^ Division of Gynecologic Oncology, Department of Obstetrics and Gynecology Washington University School of Medicine St Louis Missouri; ^7^ Division of Nuclear Medicine Edward Mallinckrodt Institute of Radiology, Washington University School of Medicine St Louis Missouri

**Keywords:** AKT, BRAF, MAPK, MEK, PPAR, RET, thyroid cancer, tissue array analysis

## Abstract

**Background:**

Prognosis among patients with differentiated thyroid cancer is widely variable. Better understanding of biologic subtypes is necessary to stratify patients and improve outcomes.

**Methods:**

In patients diagnosed with classic histology papillary thyroid cancer treated from 1973 to 2009, BRAF V600E mutation status was determined on surgical tumor specimens by restriction fragment length polymorphism analysis. A tissue microarray (TMA) was constructed from tumor specimens in triplicate and stained by immunohistochemistry for RET, phospho‐MEK, MAPK(dpERK), PPARγ, and phospho‐AKT(pAKT). Stained slides were scored independently and blindly by two investigators and compared to tumor and patient characteristics and outcomes.

**Results:**

A total of 231 patients had archived formalin‐fixed, paraffin‐embedded tumor tissue available and were included on the TMA. Mean age at diagnosis was 44 years (range 6‐82 years); proportion of patients with female sex was (72%); 2015 American Thyroid Association (ATA) risk stratification was low (26%), intermediate (32%), and high (42%). BRAF V600E mutation was found in 74% of specimens, and IHC was scored as positive for RET (61%), MAPK (dpERK) (14%), PPARγ (27%), and pAKT (39%). Positive RET staining was associated with a lower risk of recurrence (HR = 0.46, 95% CI 0.22‐0.96). No other molecular biomarkers were independent predictors of recurrence on univariable analysis. On RPA, patients with RET‐negative and either MAPK(dpERK)‐positive or pAKT‐positive tumors were identified to have a high risk of recurrence (HR = 5.4, 95%CI 2.5‐11.7). This profile remained associated with recurrence in a multivariable model including ATA risk stratification (HR = 2.8, 95% CI 1.3‐6.0).

**Conclusion:**

Characterization of molecular pathways involved in cPTC tumorigenesis may add further risk stratification for recurrence beyond the 2015 ATA risk categories alone.

## 1 INTRODUCTION

Thyroid cancer represents the most common endocrine malignancy, accounting for an estimated 53 990 new diagnoses in the United States in 2018.^1^ Papillary thyroid cancers (PTC) account for 65%‐88% of all thyroid cancers.^2^ Post‐thyroidectomy pathological findings play an important role in prognosis, and along with patient age and total body iodine uptake scan, comprise the three‐tiered risk stratification system endorsed in the American Thyroid Association (ATA) 2015 guidelines to predict risk of recurrent disease.^3^ Rates of recurrence vary widely within risk groups,^3^ reflecting residual heterogeneity. Differences in tumor biology may explain much of the variability, and molecular markers of aggressive disease have been proposed to further stratify the three‐tiered risk system.

The proto‐oncogene BRAF V600E mutation has been extensively studied and characterized in the PTC literature, though with conflicting associations with recurrence in multiple studies. A pooled meta‐analysis of 14 studies including 2470 PTC patients found BRAF mutation was associated with twice the risk of recurrence (24.9% vs 12.6%, *P* < 0.00001).^4^ It is unclear whether BRAF mutation independently confers additional risk as it is often linked to aggressive histological findings.^5^ Though BRAF mutation is quite prevalent (30%‐80%),^6^ even in the intermediate‐ and high‐risk groups, BRAF mutation alone is not consistently prognostic. In our prior institutional series of 508 PTC patients with 8‐year median follow‐up, nearly half of the patients had multifocal disease, extrathyroidal extension, and cervical nodes. BRAF V600E mutation was prevalent (67%), but was not associated with recurrence or disease‐specific survival.^7^ This suggests that additional molecular drivers may be responsible for aggressive cPTC.

The Cancer Genome Atlas (TCGA) recently reported their analysis of 496 PTC samples and found significant genetic variation amongst the BRAF mutant cohort.^8^ In their analysis, two common oncogenic pathways were identified: (a) BRAF mutation constitutively activating mitogen‐activated protein kinase kinase (MEK) and mitogen‐activated protein kinase (MAPK), and (b) RAS activating both PI3 kinase/phosphorylated AKT (pAKT) and the MAPK/Extracellular Signal‐Related Kinase (ERK) pathway. Upstream of either proposed pathway is the RET proto‐oncogene (RET). Downstream effects of cell proliferation and dedifferentiation are thought to promote tumor growth, invasiveness, and resistance to radioactive iodine.^8^, ^9^ It is possible that genetic, transcriptional, or post‐translational regulation of factors along this pathway could have similar downstream effects even in the absence of upstream genetic activation. Therefore, we sought to characterize the molecular expression at the protein level of five factors along the BRAF and RAS pathways in a large single institution cohort of PTC patients with long clinical follow‐up. We sought to identify molecular signatures that may be associated with clinical outcomes.

## 2 METHODS

### 2.1 Patient selection

Patients referred to Radiation Oncology from 1973 to 2009 with a diagnosis of classic papillary histology thyroid cancer (cPTC) who underwent thyroidectomy and had available archived tumor specimens were included. Other histologic variants of PTC, including follicular and tall cell variants, were specifically excluded. This retrospective study was approved by the institutional review board with waiver of consent.

### 2.2 BRAF V600E mutation analysis

BRAF V600E mutation status was determined as previously published.^7^ Briefly, thyroid tumor specimens were unarchived from formalin‐fixed, paraffin‐embedded (FFPE) biopsies. Tissue slides stained with hematoxylin and eosin were examined and marked by a board certified pathologist (JP) to identify areas of cPTC. For BRAF mutation status, total RNA was isolated from two tissue cores of 1 mm diameter and the BRAF exon was amplified with polymerase chain reaction (PCR). BRAF V600E mutation was identified with restriction fragment length polymorphism (RFLP) analysis, which has been previously validated.^7^

### 2.3 Immunohistochemistry analysis

A tissue microarray (TMA) was constructed from the same FFPE samples. Areas of cancer were punched with 1.5 mm cores and placed in triplicate into recipient blocks. IHC stains for RET (C‐19; Santa Cruz Biotechnology, Dallas, TX), phospho‐MEK (pMEK; 166F8; Cell Signaling Technology, Danvers, MA), phospho‐p44/42 MAPK (dpERK; Erk1/2, Thr202/Tyr204, 20G11; Cell Signaling Technology), PPARγ (SC‐7273P; Santa Cruz Biotechnology), and phospho‐AKT (pAKT; Ser473, Thr308; Cell Signaling Technology) were scored independently and blindly by two investigators on a scale of 0‐3 for staining intensity and percentage of stain positive cells. The initial scoring of 0 to 3 was chosen to better characterize the range of staining intensity/percent positive seen in the cohort. Examples of control slides staining and each score for the TMA are presented in Supplemental Figures S1 and S2, respectively. An average score of ≥2 was categorized as positive, as judged by either investigator, and <2 was categorized as negative. Concordance in scoring between both investigators was determined to document interpersonal scoring heterogeneity.

### 2.4 Treatment and follow‐up

All patients underwent thyroidectomy. Post‐operative radioactive iodine (RAI) was administered to 215 patients. Whole‐body scans were typically performed post‐RAI ablation, and surveillance total body scans were performed after thyroid hormone withdrawal at 1 year following RAI. Non‐palpable persistent disease on the whole‐body scan was treated with additional RAI. Surveillance consisted of physical examination and laboratory studies including thyroid stimulating hormone (TSH), triiodothyronine, and free thyroxine with the addition of thyroglobulin levels in the latter years of the study. Recurrence was defined as either positive disease found on the whole‐body scan in a previously negative area, positive disease seen on other clinical imaging (eg, neck ultrasound or CT scan), or pathologically proven recurrence. Metastatic patients who never responded to RAI and had continued progression of disease were also coded as recurrent at time of diagnosis.

### 2.5 Statistics

Concordance of TMA scoring was evaluated with Cohen's kappa,^10^ with a cutoff score above 0.4 required to be included in further analysis. Two‐tailed student’s *t* tests were used to compare continuous variables, and Fisher’s exact tests were used to compare categorical variables. Significant differences were considered if *P* < 0.05. Binary logistic regression was used to identify significant clinicopathologic factor associations with each molecular marker. Kaplan‐Meier and log‐rank tests were used for freedom from recurrence (FFR) and cancer‐specific survival (CSS). Univariable and multivariable Cox regression was used to model predictors of recurrence and thyroid cancer death. Results were considered significant if the 95% confidence interval (CI) did not cross one.

In order to determine molecular risk classification, recursive partition analysis (RPA) was conducted for the outcome of disease utilizing survival trees. Pruning was completed, when necessary, by recursively snipping off the least important splits based on the complexity parameter (CP). All statistical analyses were done with R version 3.03, SAS version 9.4, and SPSS, version 23 (IBM, Armonk, NY).

## 3 RESULTS

### 3.1 Patients and treatments

A total of 1712 patients with a diagnosis of thyroid cancer were referred to our department from 1973 to 2009. A total of 383 patients with cPTC histology who underwent partial or total thyroidectomy had available tumor specimens. Of these patients, 244 had adequate archived tumor specimen for molecular analyses. A total of 231/244 (95%) patients had interpretable IHC stains on the TMA and were included for analysis. The final cohort had a mean follow‐up of 10.1 years (range 0.1‐40 years).

Patient and tumor characteristics are detailed in Table 1. The majority were adult (n = 212) compared to pediatric (≤21 years of age; n = 19). Mean age for adult patients was 47 years (range 23‐82 years) compared to 16 years (range 6‐21 years) for pediatric patients. Cervical lymph node dissection was done in 49% of cases. Adjuvant I‐131 was given to 94% of patients in this study. Tumor size and nodal stage were significantly higher in pediatric patients. ATA risk groups were represented equally between adult and pediatric patients. The majority of patients had intermediate‐ (32%) or high‐risk (42%) disease.

**Table 1:** Patient and tumor characteristics

	Total (n = 231)	Adult (n = 212)	Pediatric (n = 19)	*P* value
BRAF V600E mutation	170/231 (74%)	156/212 (72%)	14/19 (74%)	1.00
Positive pAKT IHC	83/212 (39%)	75/194 (39%)	8/18 (44%)	0.62
Positive MAPK IHC	29/206 (14%)	28/188 (15%)	1/18 (6%)	0.48
Positive RET IHC	124/205 (61%)	120/189 (64%)	4/16 (25%)	0.006
Positive PPARγ IHC	53/194 (27%)	50/177 (28%)	3/17 (18%)	0.57
Male	65 (28%)	61 (29%)	4 (21%)	0.60
Female	166 (72%)	151 (71%)	15 (79%)	
White	197 (85%)	181 (85%)	16 (84%)	0.18
Black	17 (7%)	17 (8%)	0 (0%)	
Asian	13 (6%)	11 (5%)	2 (11%)	
Hispanic	4 (2%)	3 (2%)	1 (5%)	
Mean age (range)	44 (6‐82)	47 (23‐82)	16 (6‐21)	<0.001
Lymph node dissection	116 (50%)	103 (49%)	13 (68%)	0.15
Median Post‐op I‐131 dose (mean, range)	105 (0‐230)	105 (0‐230)	125 (0‐175)	0.49
Mean tumor size (range)	2.03 (0.1‐8.5)	1.99 (0.1‐8.5)	2.45 (0.8‐4.5)	0.10
T1	94 (41%)	91 (43%)	3 (16%)	0.01
T2	45 (20%)	38 (18%)	7 (37%)	
T3	23 (10%)	23 (11%)	0 (0%)	
T4	68 (29%)	59 (28%)	9 (47%)	
N0	116 (50%)	110 (52%)	6 (32%)	0.01
N1a	76 (33%)	71 (34%)	5 (26%)	
N1b	38 (17%)	30 (14%)	8 (42%)	
M0	220 (96%)	202 (96%)	18 (95%)	0.59
M1	10 (4%)	9 (4%)	1 (5%)	
AJCC stage				
I	154 (67%)	136 (65%)	18 (95%)	0.02
II	14 (6%)	13 (6%)	1 (5%)	
III	54 (24%)	54 (26%)	0 (0%)	
IV	7 (3%)	7 (3%)	0 (0%)	
Multifocal	112/230 (49%)	108/211 (50%)	9/19 (47%)	1.00
Capsular invasion	134/230 (58%)	123/211 (58%)	11/19 (58%)	1.00
Vascular invasion	34/230 (15%)	27/211 (13%)	7/19 (37%)	0.01
Soft tissue invasion	92/230 (40%)	81/211 (38%)	11/19 (58%)	0.14
Positive margins	85/230 (37%)	75/211 (36%)	10/19 (53%)	0.15
Tumor location				
Thyroid only	113 (49%)	107 (50%)	6 (32%)	0.25
Thyroid and neck nodes	107 (46%)	95 (45%)	12 (63%)	
Thyroid, nodes, and lung	9 (4%)	8 (4%)	51 (5%)	
Thyroid and bone	1 (0.5%)	1 (0.5%)	0 (0%)	
Unspecified	1(0.5%)	1 (0.5%)	0 (0%)	
ATA 2015 risk group				
Low	61 (26%)	56 (26%)	5 (26%)	0.21
Intermediate	74 (32%)	71 (34%)	3 (16%)	
High	96 (42%)	85 (40%)	11 (58%)	

AJCC, American Joint Committee on Cancer; ATA, American Thyroid Association; IHC, immunohistochemistry.

### 3.2 TMA scoring concordance

Two investigators independently scored the IHC staining on the TMAs on a scale from 0 to 3, and their average scores for the two to three cores per patient were compared. An average score greater than or equal to two from at least one investigator was considered positive. Concordance in scoring between investigators was 72%, 87%, 93%, 85%, and 88% for RET, pMEK, MAPK (dpERK), pAKT, and PPARγ, respectively. Cohen’s kappa was 0.46 (95% CI 0.38‐0.54), 0.14 (95% CI −0.09‐0.37), 0.68 (95% CI 0.57‐0.79), 0.68 (95% CI 0.61‐0.75), and 0.63 (95% CI 0.53‐0.73) for RET, pMEK, MAPK (dpERK), pAKT, and PPARγ, respectively. pMEK staining was positive in 26/184 (14%) of patients, but the two investigators only agreed on 6 of the 26 positive scores. Cohen’s kappa for MEK crossed zero, and thus, MEK staining was excluded in further analyses.

### 3.3 Association of molecular markers with clinicopathologic factors

The overall rate of BRAF V600E mutation in this population was 74%, with similar incidence in both adult and pediatric patient populations. BRAF V600E mutation was significantly enriched in the ATA intermediate‐ (73%) and high‐risk (82%) groups compared to low‐risk (61%) patients (*P* = 0.01; Table 2). BRAF V600E mutation was associated with clinical stage T3‐4 (OR 2.2, 95% CI 1.2‐4.2), capsular invasion (OR 2.0, 95% CI 1.1‐3.6), and soft tissue invasion (OR 2.3, 95% CI 1.2‐4.4). BRAF V600E mutation was inversely linked to MAPK (dpERK) IHC intensity (OR 0.43, 95% CI 0.19‐0.97), but was not associated with other molecular markers (Supplemental Table S1).

**Table 2:** Molecular marker expression in ATA low‐, intermediate‐, and high‐risk groups

	ATA low risk (%)	ATA intermediate risk (%)	ATA high risk (%)	*P* value
BRAF V600E mutation	37/61 (61)	54/74 (73)	79/96 (82)	0.01
Positive pAKT IHC	23/56 (41)	29/67 (43)	31/89 (35)	0.54
Positive MAPK IHC	9/53 (17)	10/64 (16)	10/89 (11)	0.58
Positive RET IHC	34/55 (62)	40/64 (63)	50/86 (58)	0.86
Positive PPARγ IHC	19/51 (37)	19/60 (32)	15/83 (18)	0.03

ATA, American Thyroid Association; IHC, immunohistochemistry.

The overall rate of positive RET staining was 61%. Pediatric patients had significantly lower rates of positive RET staining compared to adults (25% vs 64%, *P* = 0.002). Males also were less likely to have tumors staining positive for RET (OR 0.39, 95% CI 0.21‐0.72). There was no difference in RET staining among the ATA risk groups. RET staining did not have any association with BRAF V600E mutation, but was positively associated with pAKT and PPARγ (Supplemental Table S1).

The rates of pAKT, MAPK (dpERK), and PPARγ positive staining were 39%, 14%, and 27%, respectively. There were no significant differences between adult and pediatric patients. PPARγ positive staining was less likely in the ATA high‐risk group (Table 2). PPARγ positive tumors were less likely to be T3‐4 clinical stage (OR 0.75, 95% CI 0.58‐0.97), lymph node positive (OR 0.38, 95% CI 0.20‐0.74), multifocal (OR 0.44, 95% CI 0.23‐0.85), and less likely to have soft tissue invasion (OR 0.41, 95% CI 0.21‐0.83), or positive margins (OR 0.35, 95% CI 0.17‐0.73). pAKT, MAPK (dpERK), and PPARγ co‐expression was common (Supplemental Table S1).

### 3.4 Recurrence and cancer‐specific mortality

**Figure 1:**
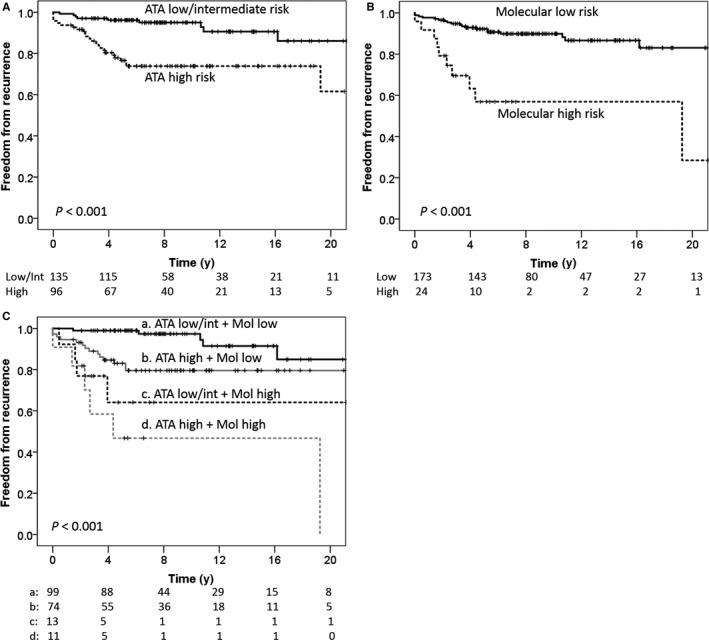
Kaplan‐Meier analysis for freedom from recurrence (FFR). A, FFR of patients stratified by ATA risk groups, B, FFR of patients stratified by a proposed molecular score. C, FFR of patients stratified by both ATA risk groups and a proposed molecular score

Recurrence occurred in 34/231 (14.7%) of cPTC patients during a mean of 10 years of follow‐up. Of these, 3 (9%) recurred in the thyroid bed alone, 23 (67%) recurred in neck lymph nodes, 4 (12%) recurred in distant sites, and 4 (12%) patients never achieved remission from distant disease since their initial surgery. Rates of recurrence in the ATA low‐, intermediate‐, and high‐risk groups were 5%, 10%, and 25%, respectively (*P* = 0.001). There was no significant difference in recurrence between ATA low‐ and intermediate‐risk groups (Table 3). However, the estimated 10‐year FFR was significantly worse in the ATA high‐risk group compared to the pooled low‐ and intermediate‐risk groups in Kaplan‐Meier analysis (74% vs 95%, *P* < 0.001, Figure 1A). RET expression was inversely associated with recurrence (HR 0.46, 95% CI 0.22‐0.96). No other molecular markers were independently associated with recurrence in this patient population (Table 3).

**Table 3:** Univariable analyses of recurrence and cancer‐specific death and their associations with molecular and clinicopathologic factors. Hazard ratios and 95% confidence intervals (CI) are noted for recurrence and cancer‐specific death

	Recurrence (Hazard ratio, 95% CI) n = 34/231	Cancer death (Hazard ratio, 95% CI) n = 9/231
BRAF V600E mutation	1.56 (0.68‐3.59)	3.48 (0.43‐27.9)
Positive pAKT IHC	1.09 (0.53‐2.25)	0.60 (0.12‐2.95)
Positive MAPK IHC	1.57 (0.65‐3.84)	0.71 (0.09‐5.70)
Positive RET IHC	**0.46 (0.22‐0.96** **)**	0.45 (0.11‐1.91)
Positive PPARγ IHC	0.37 (0.11‐1.23)	1.01 (0.21‐4.93)
Molecular high‐risk score	**5.57 (2.56‐12.1)**	**4.73 (1.12‐19.9)**
Age	1.00 (0.98‐1.03)	**1.05 (1.01‐1.10)**
Male vs female	1.47 (0.72‐2.97)	1.96 (0.52‐7.30)
Race		
White (Ref)	Ref	Ref
Black	0.38 (0.05‐2.82)	Unable to calculate HR^a^
Asian	0.99 (0.23‐4.20)	1.85 (0.23‐14.9)
Hispanic	2.03 (0.28‐14.9)	Unable to calculate HR^a^
Tumor size	**1.37 (1.18‐1.59)**	1.28 (0.95‐1.73)
T3‐4 vs T1‐2	**3.10 (1.54‐6.28)**	**12.3 (1.53‐98.3)**
Node positive	**5.25 (2.17‐12.7)**	7.85 (0.98‐62.8)
M1 vs M0	**7.47 (2.85‐19.6)**	**16.1 (3.82‐67.8)**
AJCC stage		
III‐IV vs I‐II	**3.42 (1.74‐6.72)**	**26.3 (3.26‐211)**
Multifocal	**2.29 (1.10‐4.76)**	7.80 (0.96‐63.6)
Capsular invasion	**3.06 (1.33‐7.07)**	Unable to calculate HR^b^
Vascular invasion	**5.02 (2.48‐10.2)**	**5.26 (1.31‐21.2)**
Soft tissue invasion	**3.37 (1.63‐6.95)**	**10.9 (1.34‐88.9)**
Positive margins	**2.59 (1.30‐5.18)**	**12.4 (1.52‐101)**
First I‐131 dose	1.00 (0.99‐1.01)	1.01 (0.99‐1.03)
ATA risk		
Low	Ref	Ref
Intermediate	2.01 (0.52‐7.77)	Unable to calculate HR^b^
High	**5.90 (1.78‐19.6)**	Unable to calculate HR^b^

Bolded text signified the table variable was statistically significant.

AJCC, American Joint Committee on Cancer; ATA, American Thyroid Association; IHC, immunohistochemistry; Ref, reference variable.

a No cancer death in patient group.

b No cancer death in reference group.

There were 9/231 (3.9%) deaths attributed to thyroid cancer. Rates of cancer‐specific death in the ATA low‐, intermediate‐, and high‐risk groups were 0%, 1.4%, and 8.3%, respectively (*P* = 0.01). Cancer‐specific death was significantly associated with older age, higher T stage, metastatic disease, vascular invasion, soft tissue invasion, and positive margins (Table 3). No single molecular marker was independently associated with cancer‐specific death.

### 3.5 Molecular recursive partitioning analysis

**Figure 2:**
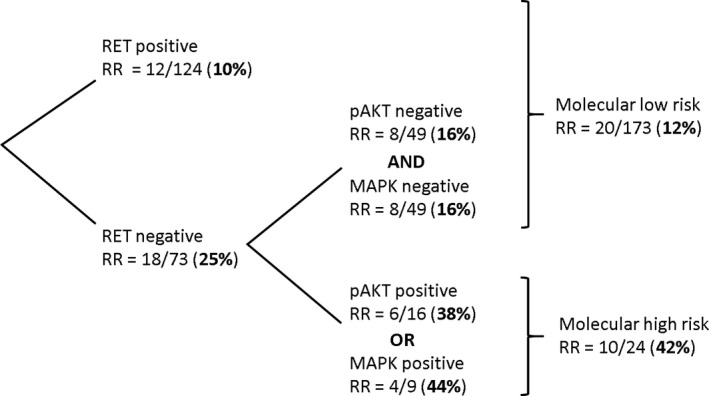
Recursive partitioning analysis of molecular markers and recurrence risk (RR). RET, pAKT, MAPK, BRAF, and PPARγ were included in the initial model, and the tree was pruned manually

In exploratory analysis, we sought to identify a molecular signature that independently predicts a high risk of recurrence. RET, the only marker independently associated with recurrence, was chosen as the initial branch point of a recursive partitioning tree. The other molecular markers (BRAF, MAPK (dpERK), pAKT, PPARγ) were tested as branch points to enrich for a “molecular high‐risk group” (Supplemental Figure S1). BRAF V600E mutation did not meet significance as a branch point in any iteration of the RPA. The final model is depicted in Figure 2. Positive RET staining was associated with a low risk of recurrence (10%). Patients whose tumors had negative RET staining and either positive pAKT or MAPK (dpERK) staining had a high risk of recurrence (42%). Patients whose tumors were negative for RET, pAKT, and MAPK (dpERK) had a 16% risk of recurrence and were grouped with the patients whose tumors stained positive for RET as part of a “molecular low‐risk group.” The estimated 10‐year freedom from recurrence was significantly lower in the molecular high‐risk group compared to the molecular low‐risk group (57% vs 90%, *P* < 0.001, Figure 2B). Incidence of cancer‐specific death in the molecular low‐risk and molecular high‐risk groups was 3% and 13%, respectively (*P* = 0.03).

Incidence of both BRAF mutation and PPARγ expression was not different between the low‐risk and high‐risk molecular score groups (BRAF: 73% vs 75%, respectively, *P* = 0.82; PPARγ: 27% vs 30%, respectively, *P* = 0.70). The presence of BRAF V600E mutation was not associated with risk of recurrence in either the molecular low‐risk (log‐rank *P* = 0.34) or high‐risk groups (log‐rank *P* = 0.16).

### 3.6 Comparison of ATA risk and molecular score risk

The molecular high‐risk profile was found in 9%, 14%, and 13% of ATA low‐, intermediate‐, and high‐risk groups, respectively (*P* = 0.77), suggesting the prognostic information gained from each of the two risk stratifications is unique. In a multivariable model, recurrence was independently associated with both ATA high risk (HR = 2.8, 95% CI 1.3‐6.0, *P* = 0.008) and the molecular high‐risk signature (HR = 5.4, 95% CI 2.5‐12, *P* < 0.001). Cancer‐specific death was also independently associated with both ATA high risk (HR = 9.3, 95% CI 1.1‐76, *P* = 0.04) and the molecular high‐risk signature (HR = 4.3, 95% CI 1.0‐18, *P* = 0.05). The 5‐year estimated FFR in ATA low‐/intermediate‐risk patients stratified by molecular risk was 99% (molecular low risk) vs 64% (molecular high risk), *P* < 0.001 (Figure 1C). The 5‐year estimated FFR in ATA high‐risk patients stratified by molecular risk was 83% (molecular low risk) vs 47% (molecular high risk), *P* < 0.001 (Figure 1C).

## 4 DISCUSSION

This large retrospective study of cPTC patients at a single institution characterized the expression of factors upstream and downstream along the BRAF/MAPK/ERK and RAS/pAKT pathways. We observed differences in RET tumor staining between adult and pediatric patients, and differences in BRAF V600E mutation between ATA risk groups. Clinical follow‐up of up to 20 years in this cohort allowed adequate time for recurrences and cancer‐specific survival to be assessed. As expected, recurrence was more likely in the ATA high‐risk group (25%). Only 5% and 10% of the ATA low‐ and intermediate‐risk patients recurred, respectively, which is comparable to prior reports.^3^ With the exception of RET expression, the molecular markers individually did not associate with recurrence risk. However, a proposed molecular risk stratification was identified from recursive partitioning analysis of multiple molecular markers. Patients whose tumors displayed the molecular high‐risk group of RET‐negative and either pAKT‐positive or MAPK (dpERK)‐positive IHC had quadruple the risk of recurrence compared to the low‐risk group. The molecular risk score also assigned risk of recurrence independent of classic pathologic findings, suggesting molecular subtypes of cPTCs may explain some of the heterogeneity seen in outcomes based on the 2015 ATA risk classification system.

This study incorporates immunohistochemical staining of multiple molecular markers, including RET, into a prognostic score for disease recurrence in cPTCs ranging from ATA low to high risk. Prior efforts to incorporate molecular prognostics have focused on tumor genetic mutations. Genetic panels are being developed to differentiate benign from malignant thyroid nodules.^11^ Niemeier et al created a model utilizing BRAF mutation and clinicopathologic factors for predicting extrathyroid tumor spread in papillary microcarcinomas.^12^ Most recently, a mutation in the telomerase reverse transcriptase (TERT) promoter with or without a concomitant BRAF V600E mutation was found to be associated with recurrence and cancer death risk.^13^, ^14^ Several other prognostic score algorithms have been suggested, but these have not incorporated molecular analysis of tumors.^15^ An advantage of IHC over genetic analysis alone is that translated protein products and post‐translational regulatory modifications such as activating phosphorylation of kinases can be qualitatively assessed.

Similar to our prior report,^7^ in this cohort, BRAF V600E mutation was not associated with recurrence or death. Rather, MAPK (dpERK) staining in the absence of RET staining was highly associated with recurrence and death. MAPK (dpERK)/ERK is a kinase activated downstream of both the BRAF and RAS pathways, and participates in proliferation and dedifferentiation.^16^ Intriguingly, positive IHC staining of MAPK (dpERK) in BRAF mutant tumors was half as likely as in BRAF wild‐type tumors in this study. Activated pMEK staining was also significantly lower (14%) in this population than the percentage of BRAF mutants (74%) would suggest. This finding has been observed in human PTC samples^17^ as well as in melanoma, atypical nevi, and common nevi.^18^ Zuo et al showed in 42 human papillary thyroid cancer samples that only 7.1% of PTCs with BRAF mutation had activated MAPK (dpERK)/ERK. In contrast, 29% of wild‐type BRAF PTCs had activated MAPK (dpERK). In their cohort, BRAF V600E mutation was not associated with any negative clinicopathologic factors except for age >45 years.^17^ These data corroborate findings in a large 459 patient study from the University of California, San Francisco, in which BRAF V600E mutation was also not associated with negative clinicopathologic factors.^19^ In a recent publication, BRAF V600E mutation did not worsen cancer‐specific mortality in patients <45 years old.^20^ Thus, the association of BRAF V600E mutation with aggressive PTC pathology and clinical recurrence remains unclear. The lack of association between BRAF mutation and MAPK (dpERK) activation at the protein level in PTCs could partially explain this discrepancy.

In this study, patients with RET positive tumor staining alone were half as likely to experience recurrence compared to patients with RET‐negative tumors. In contrast, a meta‐analysis of eight studies with 1000 patients found RET amplification through rearrangement was associated with twice the risk of distant metastasis.^14^ RET rearrangement involving the C‐terminus tyrosine kinase domain of the protein results in constitutive activation, and these fusion proteins are detected by the C‐terminal specific antibodies commonly used for IHC, including the one used in this study.^21^, ^22^ However, the downstream phenotype of RET rearrangement depends on the specific N‐terminal fusion partner. The RET/PTC3 rearrangement, mostly found in tumors from patients with prior radiation exposure,^23^, ^24^ is associated with aggressive features such as larger tumor size, extrathyroidal extension, and nodal metastases. On the other hand, RET rearrangements in most sporadic PTCs are the RET/PTC1 rearrangement. These well‐differentiated PTCs rarely dedifferentiate and tend to have a more indolent course.^21^, ^22^ Therefore, in patients without prior radiation exposure, RET activation alone appears to be a positive prognostic biomarker. This study is limited since we did not test for RET gene rearrangement subtype, but only 7 (3%) patients were known to have had prior neck radiation exposure.

How RET rearrangement might abrogate the downstream effects of MAPK (dpERK)‐ or pAKT‐expressing tumors remains to be elucidated. In our population, 13% and 49% of samples staining positive for RET overexpressed MAPK (dpERK) and pAKT, respectively. Despite activation of the traditional BRAF/MAPK (dpERK) and RAS/pAKT pathways, these tumors behaved indolently, with only a 10% recurrence rate in our cohort. RET kinase activity may be important in maintaining differentiation through micro‐ribonucleic acid (miRNA) regulation. Prior work has shown the importance of miRNA in silencing target genes.^25^ RET mutations upregulate the biogenesis of miRNAs,^26^ and emerging work has identified miR30a as important in maintaining PTC differentiation through the inhibition of lysyl oxidase.^27^, ^28^ In addition, pAKT localization has been shown to be dependent on the presence of RET rearrangement.^29^ In non‐invasive areas of PTC co‐expressing RET, pAKT expression was diffuse and cytoplasmic. This was in contrast to invasive areas of PTC without RET co‐expression, where pAKT expression was focal and localized in the nucleus.^29^ pAKT expression in our cohort was predominantly localized in the nucleus, independent of RET staining. As 50% of the PTC patients in the previous work by Vasko et al^29^ were exposed to the Chernobyl radiation accident, differences in the specific RET rearrangement (eg, RET/PTC1 vs RET/PTC3) may account for this discrepancy. Further work is needed to study these post‐translational interactions.

Limitations of this single institution study include the retrospective bias of patient selection/referral patterns. By omitting patients not referred for adjuvant RAI, the results are potentially skewed to higher risk patients. However, patients in this cohort were treated with consistent institutional guidelines and most had long‐term follow‐up. Second, the selection of tested molecular markers in this study was decided before the TCGA analysis was published, and RAS and TERT mutations were not evaluated. These should be taken into account in follow‐up studies. Third, sampling effect and tumor heterogeneity can complicate TMA analysis of potential biomarkers, but triplicate cores and multi‐investigator blinded analysis are known to maximize consistency.^30^ Fourth, the evaluation of phosphorylated proteins by immunohistochemistry is dependent on time to tissue fixation and other conditions ex vivo which may change the phosphorylation state of the sample.^31^, ^32^ Finally, the proposed molecular risk score was derived statistically from recursive partitioning analysis and is only hypothesis‐generating. Further mechanistic work should be supported, and prognostic scores need to be validated.

## 5 CONCLUSIONS

In this exploratory analysis, positive RET staining by IHC was associated with a low risk of recurrence and death from papillary thyroid cancer. In patients with RET‐negative tumors, positive pAKT or MAPK (dpERK) staining constituted a high molecular risk subgroup of tumors. IHC analysis of molecular biomarkers may assist clinicians in further stratifying risk of recurrence when combined with the current ATA risk classifications.

## CONFLICTS OF INTEREST

The authors report no conflict of interests with this work.

## AUTHOR CONTRIBUTIONS

Alexander J. Lin: data curation, formal analysis, investigation, methodology, validation, visualization, writing—original draft, and writing—review and editing. Pamela Samson: formal analysis, methodology, validation, writing—review and editing. Todd DeWees: formal analysis, methodology, validation, writing—review and editing. Lauren Henke: data curation, writing—review and editing. Thomas Baranski: writing—review and editing. Julie Schwarz: writing—review and editing. John Pfeifer: conceptualization, data curation, formal analysis, funding acquisition, investigation, methodology, project administration, resources, software, supervision, validation, visualization, writing—original draft, and writing—review and editing. Perry Grigsby: data curation, investigation, project administration, resources, writing—review and editing. Stephanie Markovina: conceptualization, data curation, investigation, methodology, project administration, resources, supervision, validation, visualization, writing—original draft, and writing—review and editing.
