# Supplementary figures and images for: A molecular approach combined with American Thyroid Association classification better stratifies recurrence risk of classic histology papillary thyroid cancer
Source: Cancer Med. 2018 Dec 14;8(1):437–46. doi: 10.1002/cam4.1857 (PMC6346248; doi:10.1002/cam4.1857)

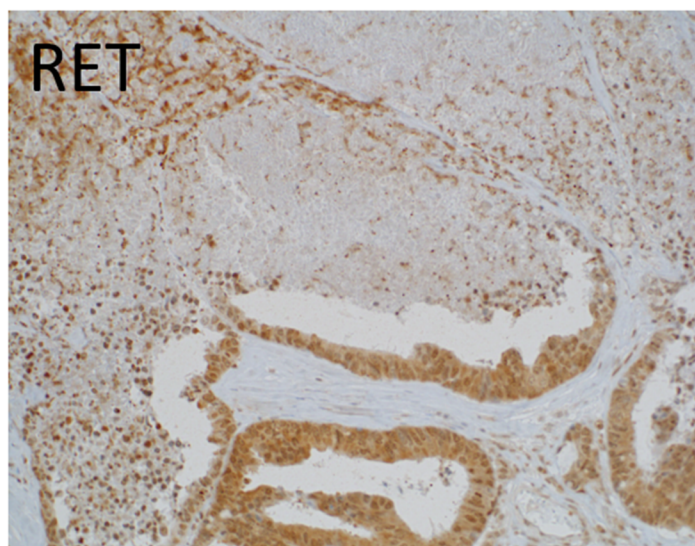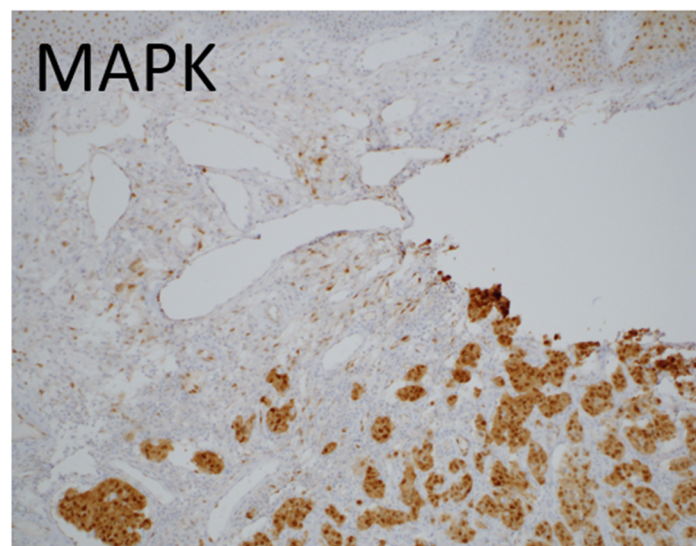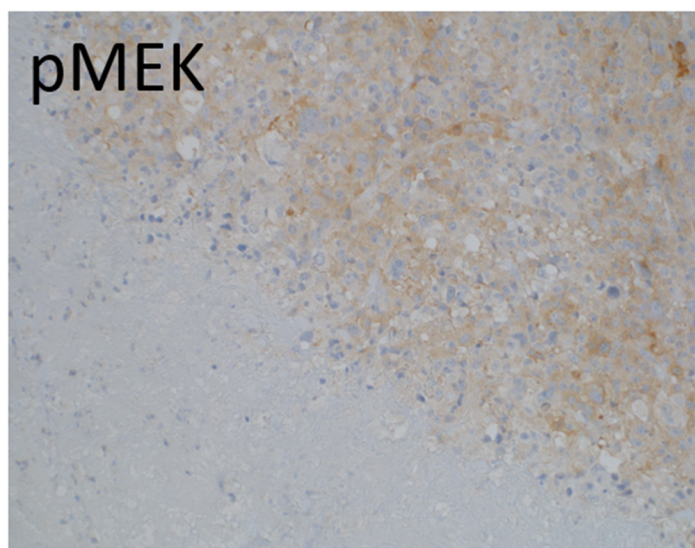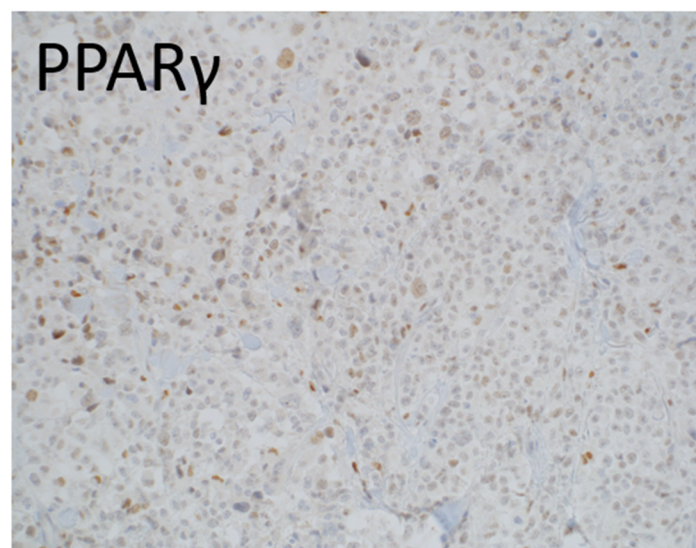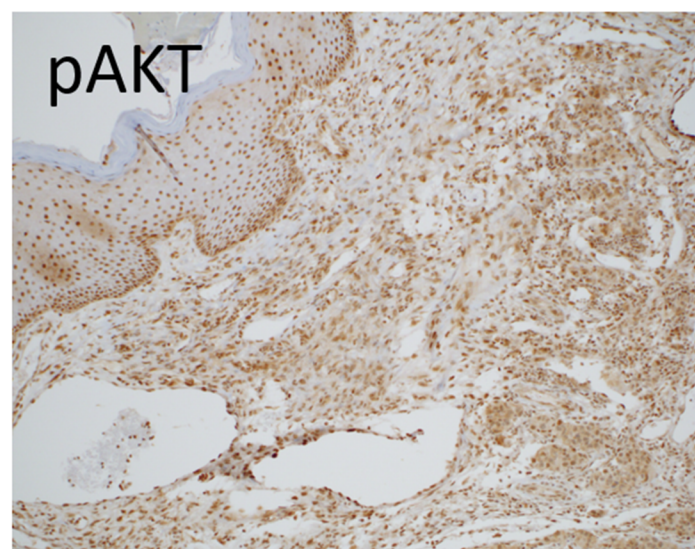

Supplement: Supplementary file 1 [file CAM4-8-437-s001.pdf]

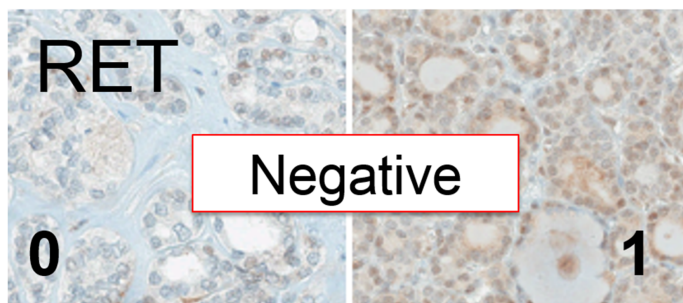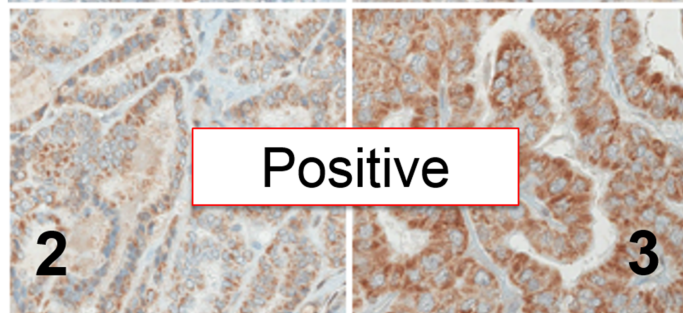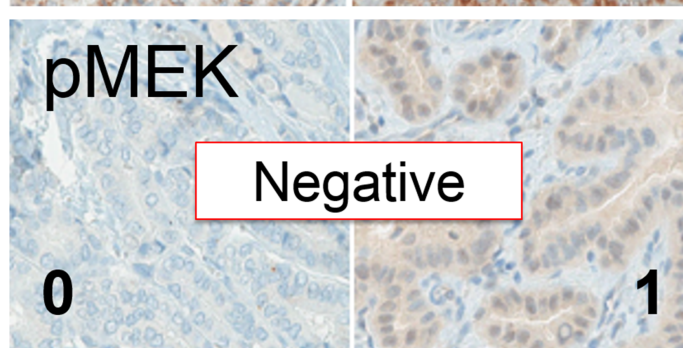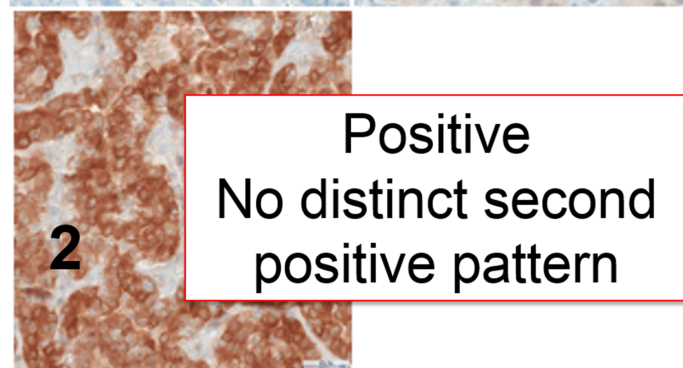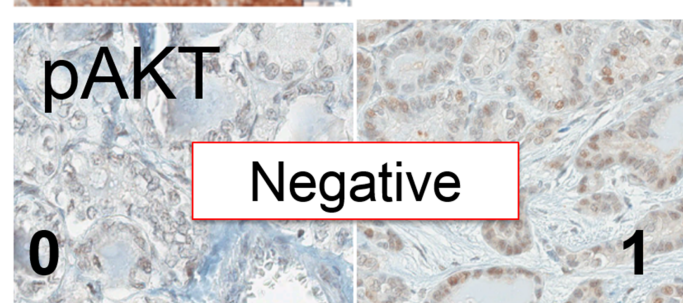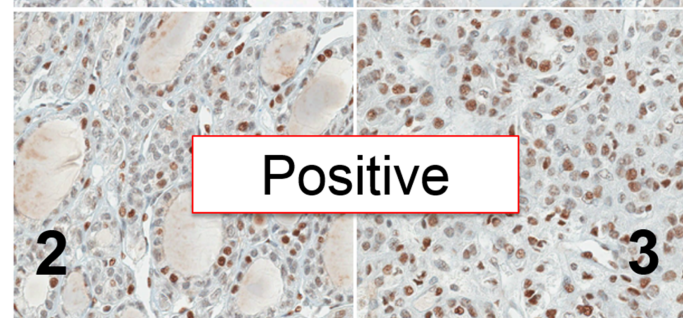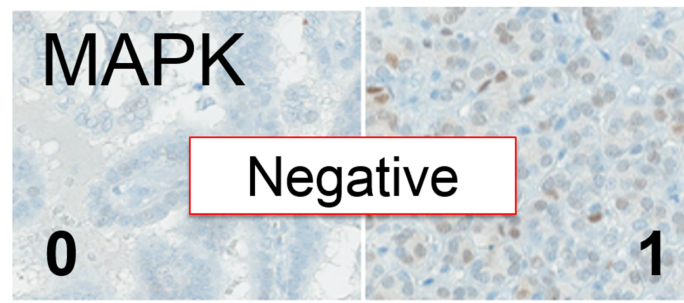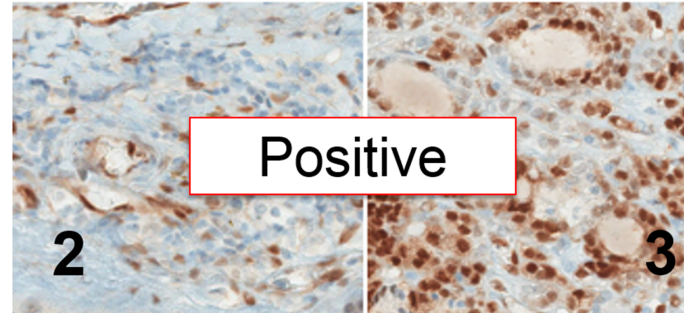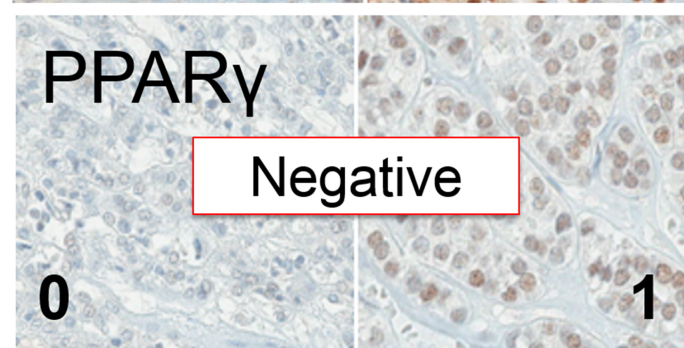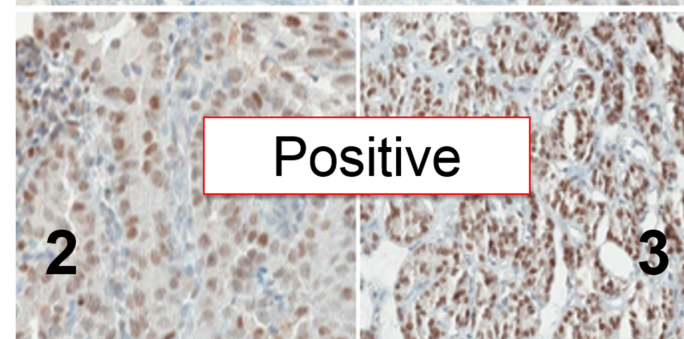

Supplement: Supplementary file 2 [file CAM4-8-437-s002.pdf]

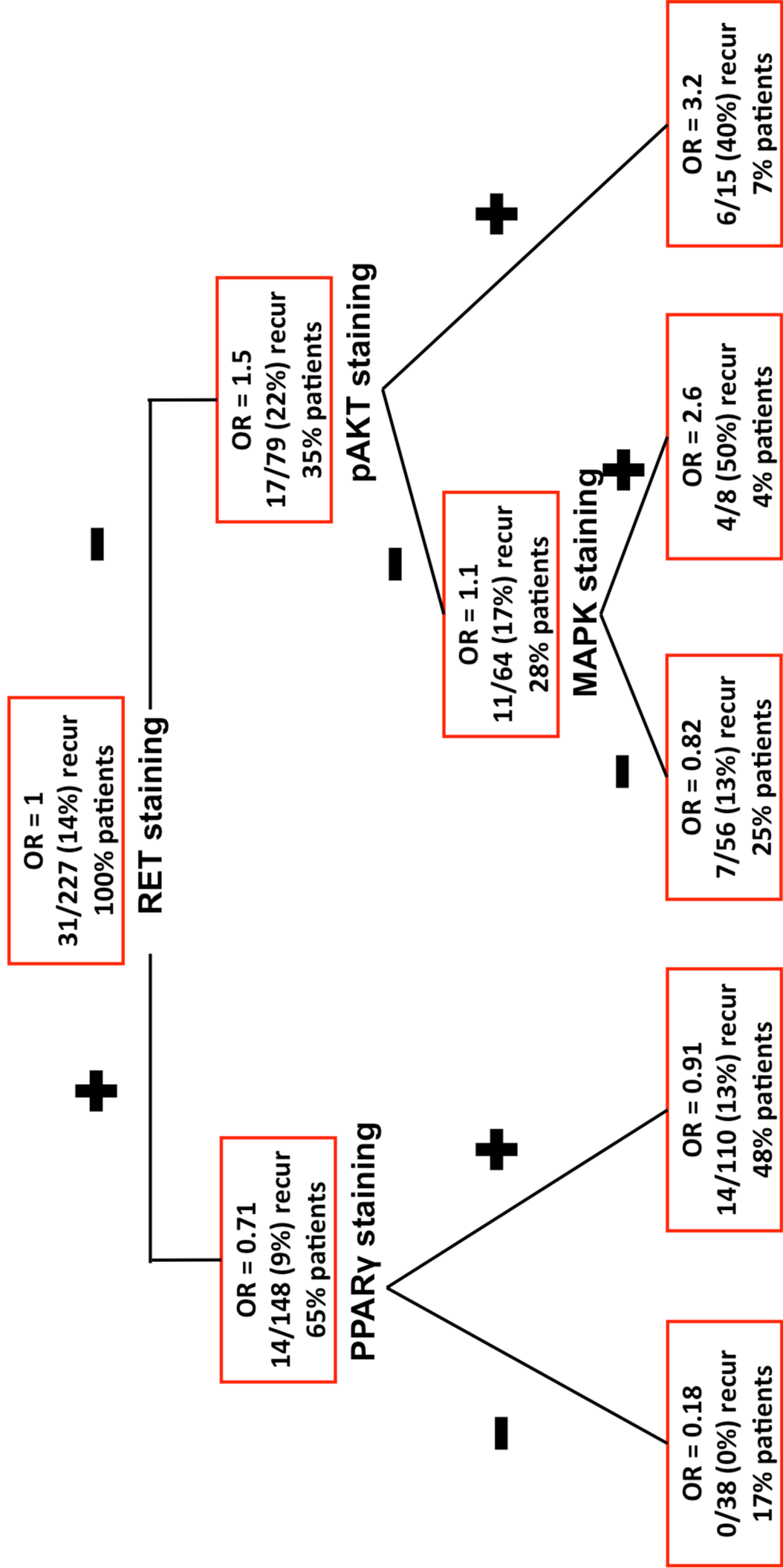

Supplement: Supplementary file 3 [file CAM4-8-437-s003.pdf]

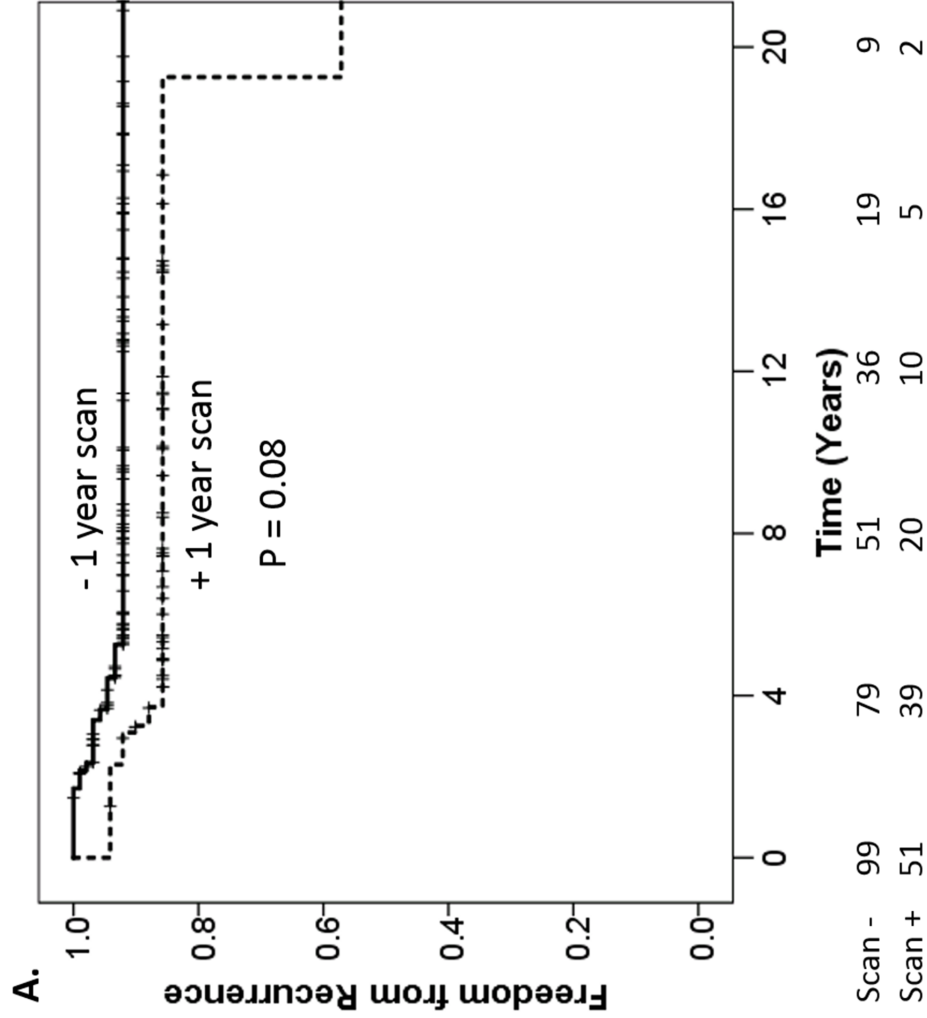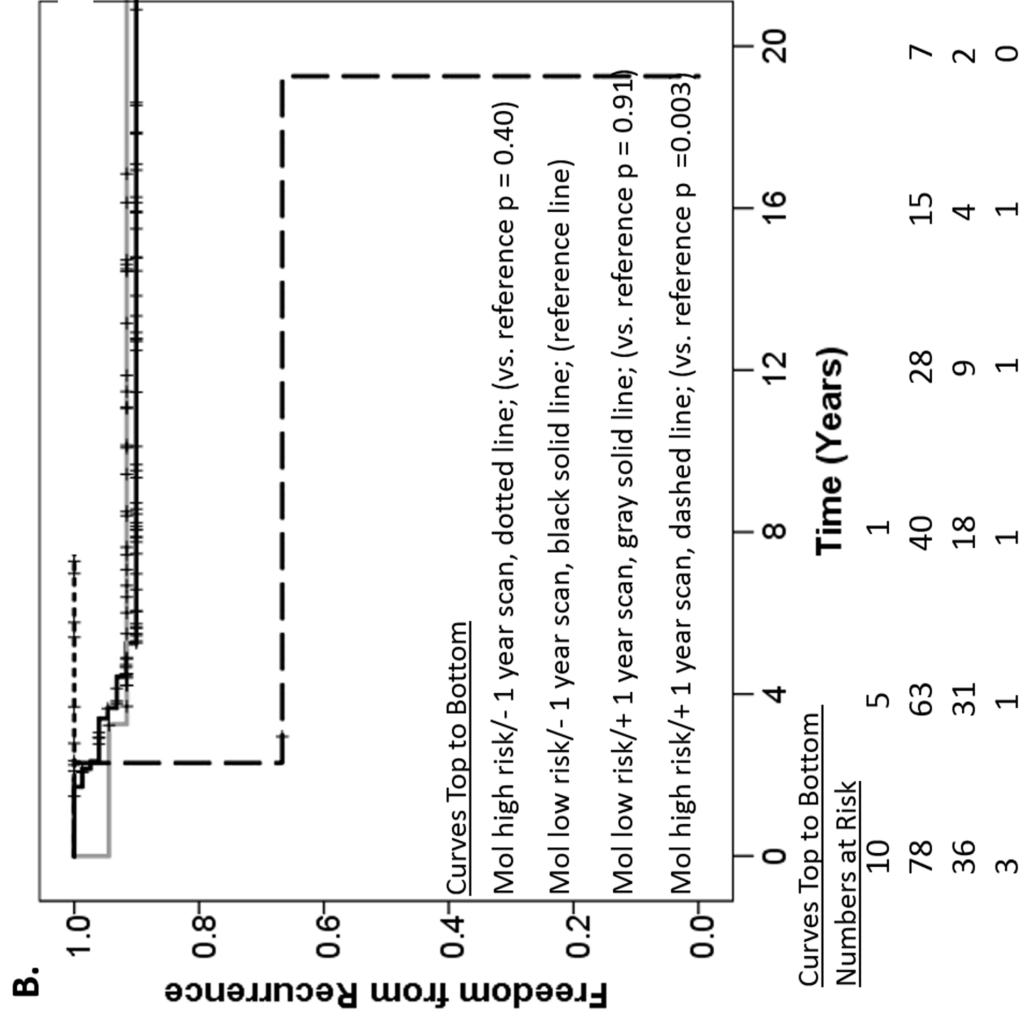

Supplement: Supplementary file 4 [file CAM4-8-437-s004.pdf]
