# Supplementary material for: A molecular approach combined with American Thyroid Association classification better stratifies recurrence risk of classic histology papillary thyroid cancer
Source: Cancer Med. 2018 Dec 14;8(1):437–46. doi: 10.1002/cam4.1857 (PMC6346248; doi:10.1002/cam4.1857)
Supplement: Supplementary file 5 [file CAM4-8-437-s005.docx]

**Supplemental Table 1:** Clinical and pathologic associations with molecular markers. Odds ratios (OR) with the 95% confidence interval are noted in parentheses.

|  | BRAF V600E mutation (170/231, 74%) | Positive pAKT IHC (83/212, 39%) | Positive MAPK IHC (29/206, 14%) | Positive RET IHC (124/205, 61%) | Positive PPARγ IHC (53/194, 27%) |
| --- | --- | --- | --- | --- | --- |
| Age | 1.00 (0.98-1.02) | 0.99 (0.97-1.01) | 1.00 (0.97-1.03) | **1.03 (1.01-1.05)** | 1.00 (0.98-1.02) |
| Male vs female | 1.02 (0.53-1.95) | 0.53 (0.28-1.02) | 0.63 (0.24-1.63) | **0.39 (0.21-0.72)** | 0.69 (0.34-1.42) |
| White race (Ref)  Non-white race | Ref  1.20 (0.51-2.81) | Ref  1.54 (0.70-3.39) | Ref  1.59 (0.59-4.28) | Ref  1.45 (0.64-6.67) | Ref  1.68 (0.74-3.82) |
| Tumor size  T3-4 vs. T1-2 | 0.95 (0.80-1.13)  **2.22 (1.16-4.24)** | 1.16 (0.98-1.38))  0.77 (0.43-1.36) | 0.90 (0.69-1.17)  0.60 (0.26-1.39) | 1.00 (0.84-1.20)  1.03 (0.58-1.83) | **0.75 (0.58-0.97)**  **0.34 (0.17-0.71)** |
| Node positive | 1.44 (0.80-2.61) | 1.04 (0.60-1.81) | 0.80 (0.37-1.77) | 0.64 (0.36-1.12) | **0.38 (0.20-0.74)** |
| M1 vs M0 | 0.84 (0.21-3.34) | 3.92 (0.98-15.6) | 1.56 (0.31-7.72) | 0.87 (0.19-4.01) | 1.09 (0.20-5.79) |
| AJCC stage  III-IV vs I-II | 1.45 (0.72-2.92) | 0.80 (0.42-1.52) | 0.41 (0.13-1.23) | 1.33 (0.70-2.54) | 0.64 (0.30-1.36) |
| Multifocal  Capsular invasion  Vascular invasion  Soft tissue invasion  Positive margins | 0.57 (0.31-1.03)  **1.98 (1.10-3.59)**  1.82 (0.71-4.64)  **2.30 (1.21-4.39)**  1.57 (0.84-2.95) | 0.70 (0.40-1.22)  0.86 (0.49-1.51)  1.27 (0.59-2.72)  0.65 (0.37-1.16)  0.85 (0.48-1.51) | 1.55 (0.70-3.44)  0.59 (0.27-1.29)  1.20 (0.42-3.43)  0.59 (0.26-1.37)  0.47 (0.19-1.16) | 0.64 (0.36-1.13)  0.86 (0.49-1.53)  0.47 (0.22-1.02)  0.78 (0.44-1.39)  1.00 (0.56-1.80) | **0.44 (0.23-0.85)**  0.65 (0.34-1.23)  0.74 (0.30-1.82)  **0.41 (0.21-0.83)**  **0.35 (0.17-0.73)** |
| BRAF V600E mutation  Positive pAKT IHC  Positive MAPK IHC  Positive RET IHC  Positive PPARγ IHC | ----  0.66 (0.36-1.23)  **0.43 (0.19-0.97)**  1.26 (0.68-2.36)  1.05 (0.51-2.19) | 0.66 (0.36-1.23)  ----  **2.22 (0.99-5.00)**  **3.68 (1.90-7.12)**  **2.81 (1.42-5.56**) | **0.43 (0.19-0.97)**  **2.22 (0.99-5.00)**  ----  1.09 (0.45-2.64)  **2.65 (1.10-6.42)** | 1.26 (0.68-2.36)  **3.68 (1.90-7.12)**  1.09 (0.45-2.64)  ----  **2.19 (1.08-4.43)** | 1.05 (0.51-2.19)  **2.81 (1.42-5.56)**  **2.65 (1.10-6.42)**  **2.19 (1.08-4.43)**  ---- |

Abbreviations: Ref- reference; IHC – immunohistochemistry; AJCC – American Joint Committee on Cancer; ATA – American Thyroid Association
